# Thrombotic microangiopathy–like acute kidney injury revealing a germline *GATA1* mutation in a child with inherited cytopenia

**DOI:** 10.1007/s00467-026-07336-0

**Published:** 2026-05-18

**Authors:** Amal E. Gohary, Doaa M. Youssef, Diana Hanna, Mona Hamed Gehad

**Affiliations:** Department of Pediatrics, Faculty of Medicine, Zagazig University, Zagazig City, Sharkia Governorate Egypt

**Keywords:** GATA1, Inherited cytopenias, Acute kidney injury, TMA‑like syndrome, Whole‑exome sequencing

## Abstract

Thrombotic microangiopathy (TMA) is characterized by microangiopathic hemolytic anemia, thrombocytopenia, and organ injury, most commonly acute kidney injury (AKI). It may occur as a primary complement‑mediated disorder or secondary to other diseases. Germline mutations in *GATA1* cause X‑linked thrombocytopenia with or without dyserythropoietic anemia. We report an 8‑year‑old boy with long‑standing steroid‑dependent cytopenias and congenital anomalies who presented with severe infection, hemolysis, thrombocytopenia, and AKI requiring hemodialysis. Although the presentation fulfilled criteria for suspected TMA, ADAMTS13 activity, complement factor H levels, and anti-factor H antibodies were normal, excluding primary TMA. Whole‑exome sequencing identified a hemizygous pathogenic *GATA1* variant (c.646C>T; p.Arg216Trp), establishing GATA1‑related dyserythropoietic anemia with thrombocytopenia. Renal function fully recovered following supportive therapy, immunomodulation, and dialysis discontinuation. This case underscores the importance of considering secondary TMA‑like syndromes in inherited marrow disorders and highlights the diagnostic value of genomic testing in atypical TMA presentations.

## Background

Germline mutations in *GATA1* disrupt erythroid and megakaryocytic differentiation and can mimic immune cytopenias. Thrombotic microangiopathy (TMA) is a clinicopathologic syndrome defined by microangiopathic hemolytic anemia, thrombocytopenia, and organ injury, commonly involving the kidney. Distinguishing primary from secondary TMA is essential for management.

## Case presentation

**Fig. 1:**
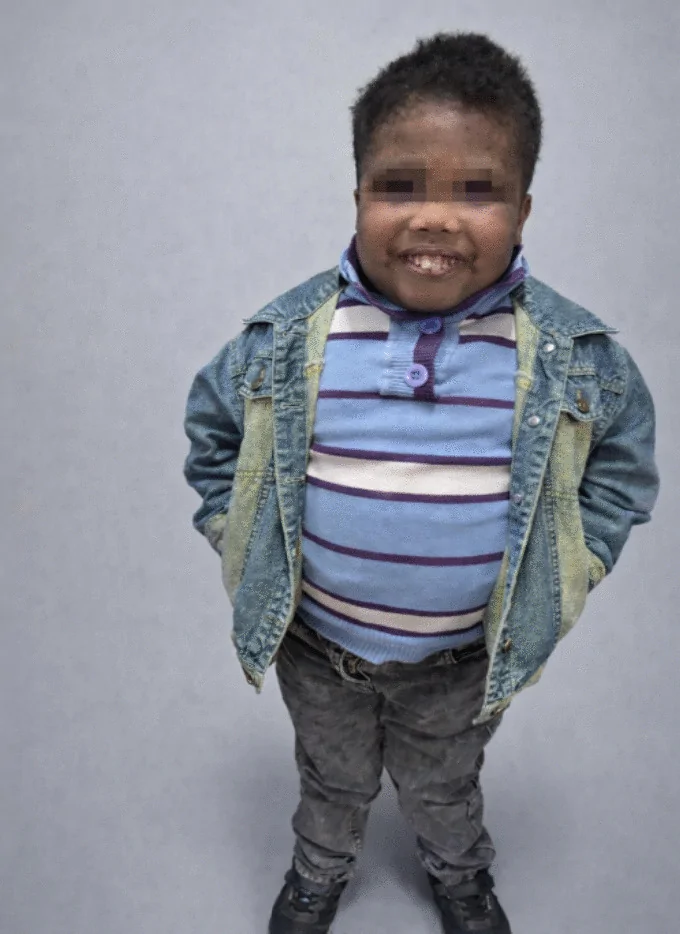
Clinical photograph of the patient showing short stature and syndromic appearance, with visible limb abnormalities

An 8‑year‑old male, the fourth child of a non‑consanguineous couple, had multiple congenital anomalies including congenital heart disease and skeletal anomalies (anonychia of the terminal phalanges) (Fig. 1). Disorders of sex development included micropenis and bilateral undescended testes with a 46,XY karyotype. At 6 months, he developed anemia requiring transfusions followed by thrombocytopenia; bone marrow biopsy done at age of 1 year old was hyper-cellular with atypical lymphoid/immune infiltration and DAT positive. Evans syndrome was diagnosed and treated with steroids for 6 years without transfusions. At 8 years, he presented with respiratory distress and epistaxis; chest radiography showed bronchopneumonia. Labs revealed that severe anemia, profound thrombocytopenia, blood film showed fragmented RBCs (schistocytes 3–4%) with high LDH 3100 U/L (reference 220–480 U/L) and reticulocytosis 3.7% compatible with microangiopathic hemolytic anemia (MAHA), and acute kidney injury (AKI) (creatinine 6 mg/dL; BUN 127 mg/dL), complicated by uremic encephalopathy requiring hemodialysis.

Further testing showed a positive Coombs test, a high reticulocyte count of 3.7%, and consumed C3 79 mg/dL (reference 90–180 mg/dL) and C4 18 mg/dL (reference 20–50 mg/dL), which normalized later in follow-up with otherwise normal immunology/virology. TMA workup (factor H, anti-factor H antibodies, ADAMTS13) was normal. Pelvi-abdominal ultrasound demonstrated bilaterally normal-sized kidneys with increased cortical echogenicity and partially preserved corticomedullary differentiation, without evidence of obstruction and mild hepatosplenomegaly.

He received hemodialysis, antibiotics, and high‑dose corticosteroids; thrombocytopenia proved steroid‑refractory but responded to IVIG. Dialysis was discontinued after nine sessions; serum creatinine normalized to 0.5 mg/dL, C3 to 99 mg/dL, C4 to 21 mg/dL and reticulocytic count to 1.9%. The patient was subsequently maintained on steroid therapy.

Although the presentation fulfilled the clinical triad of TMA, including MAHA and thrombocytopenia with AKI, normal ADAMTS13 activity and complement studies argued against primary/complement‑mediated TMA. The rapid renal recovery further argued against intrinsic microvascular renal disease. The AKI likely reflected a multifactorial process including sepsis‑associated injury, hemolysis‑related tubular toxicity, endothelial dysfunction, and hemodynamic instability.

Our patient had dysmorphic features, a syndromic presentation, short stature, stunted growth for age (Fig. 1), disordered sex development, congenital heart disease, skeletal deformity, and a complex hematological picture and TMA-like presentation in which renal biopsy was contraindicated because of persistent profound thrombocytopenia and active bleeding risk. Genetic testing was recommended.

### Genetic results

Whole‑exome sequencing (3billion EVIDENCE v4.3) identified a hemizygous likely-pathogenic *GATA1* variant: NM_002049.4:c.646C>T (NP_002040.1:p.Arg216Trp), genomic coordinate X‑48792370‑C‑T (GRCh38). Variants at the same codon have been associated with X‑linked thrombocytopenia with or without dyserythropoietic anemia (OMIM: 300367).

## Discussion

GATA1-related cytopenias (OMIM #300367) classically manifest as X-linked thrombocytopenia (XLT), with or without dyserythropoietic anemia (DA) [1, 2]. Because patients often present with macrothrombocytopenia, bone marrow dysplasia, and occasionally positive direct antiglobulin testing, the condition may be misdiagnosed as primary immune thrombocytopenia (ITP) or Evans syndrome [3].

Our case illustrates this diagnostic challenge. The patient presented in infancy with steroid-responsive cytopenias and was managed as Evans syndrome for several years. However, the presence of syndromic features, chronic disease course, and eventual identification of a pathogenic *GATA1* variant support a unifying genetic diagnosis, with the Evans syndrome-like presentation representing phenotypic overlap rather than a distinct autoimmune disorder.

Previously reported pediatric cases demonstrate overlapping but incomplete phenotypes. Nichols et al. reported the nearby p.Arg216Gln mutation associated with isolated XLT [4]. Freson et al. described the identical p.Arg216Trp variant in a neonate initially diagnosed with ITP that evolved into Evans syndrome-like cytopenias, but without AKI or syndromic features [5]. More recently, Aktekin et al. described a novel *GATA1* variant presenting as steroid-responsive ITP, contrasting with our patient’s partial response requiring IVIG [6]. Additional cases confirmed disruption of the C-terminal zinc finger domain and impaired DNA binding [7, 8].

The AKI in our patient presented with baseline anemia and  thrombocytopenia related to *GATA1* mutation complicates the interpretation of acute TMA criteria.

While the clinical triad mirrored primary TMA, the constellation of sepsis, GATA1-driven baseline cytopenia, and rapid recovery suggests that the AKI was a multifactorial “TMA-like” event rather than a primary microvascular endotheliopathy, laboratory assessment excluded ADAMTS13 deficiency and complement-mediated TMA. We therefore interpret this episode as a MAHA-associated AKI with TMA-like features, rather than definitive TMA. Secondary TMA-like syndrome triggered by severe infection and hemolysis in the context of underlying GATA1-related bone-marrow failure. Hemolysis-associated tubular injury, systemic inflammatory activation, and transient endothelial dysfunction are plausible contributors. This case highlights that inherited cytopenias may represent an unrecognized risk background for TMA-like AKI during infectious or inflammatory stress. The rapid renal recovery, absence of recurrence over 6 months of follow-up and lack of persistent renal abnormalities argue against primary complement-mediated TMA and support a secondary, transient TMA-like process [9]. While genetic testing did not identify mutations associated with atypical hemolytic uremic syndrome, we acknowledge that this does not fully exclude complement-mediated disease. However, the clinical course strongly supports a secondary, transient process.

To our knowledge, this is the first reported case of *GATA1* deficiency complicated by severe AKI requiring dialysis, with subsequent recovery following hemodialysis and immunomodulatory therapy. The AKI observed in this patient is most plausibly explained by a multifactorial process involving sepsis-associated kidney injury, hemolysis-mediated tubular toxicity, ischemic injury due to hemodynamic instability, and systemic inflammatory activation. The absence of persistent renal dysfunction (no microscopic hematuria or proteinuria) argues against congenital renal structural abnormalities or primary genetic nephropathy.

The presence of syndromic features, including short stature (SDS −2.8), 46,XY disorders of sex development with cryptorchidism and micropenis, ventricular septal defect (VSD) and brachydactyly—is atypical for isolated *GATA1* mutations. Array-comparative genomic hybridization excluded Xp11.23 microdeletions (including genes such as *SHROOM4* and *CLCN5*, which are implicated in short stature and Dent disease) [10]. Cryptorchidism is more commonly associated with alterations in *GATA4* or adjacent loci rather than *GATA1*. Growth impairment may reflect chronic anemia, prolonged corticosteroid exposure, or an additional unrecognized genetic contributor (e.g., *NSD1* overlap). Clinically, refractory early-onset Evans syndrome with dysmorphic or syndromic features should prompt genomic evaluation [10]. Future recommendations for ongoing surveillance will include serial bone marrow evaluation, endocrine assessment, and family segregation analysis.

### Limitations

We acknowledge that infection-triggered complement-mediated TMA (aHUS) cannot be definitively excluded in the absence of comprehensive complement genetic testing.

## Summary

### What is new?

- *GATA1*‑related inherited cytopenia can present with a severe TMA‑like episode requiring dialysis.
- Secondary TMA‑like syndromes should be considered in inherited marrow disorders.
- Genetic evaluation clarifies causes of atypical TMA‑like presentations.

## Data Availability

Data are available upon reasonable request from the corresponding author.
